# Cost-effectiveness of a faecal calprotectin-guided referral strategy for children with chronic abdominal symptoms: a cluster randomised controlled trial

**DOI:** 10.3399/BJGP.2025.0334

**Published:** 2026-04-08

**Authors:** Sophie M Ansems, Marjolein Y Berger, Patrick F van Rheenen, Karin M Vermeulen, Michiel R de Boer, Gea A Holtman

**Affiliations:** 1 Department of Primary and Long-term Care, University Medical Center Groningen, University of Groningen, Groningen, the Netherlands; 2 Department of Paediatric Gastroenterology, University Medical Center Groningen, University of Groningen, Groningen, the Netherlands; 3 Department of Epidemiology, University Medical Center Groningen, University of Groningen, Groningen, the Netherlands

**Keywords:** child, faecal calprotectin, functional gastrointestinal disease, inflammatory bowel diseases, primary health care

## Abstract

**Background:**

Triaging children with chronic abdominal symptoms who might benefit from paediatric specialist care is challenging for GPs.

**Aim:**

To evaluate the (cost) effectiveness of faecal calprotectin (FCal) testing to guide referral to specialist care in children with chronic abdominal symptoms.

**Design & setting:**

This pragmatic, cluster-randomised controlled trial with 1:1 randomisation of Dutch GP practices was conducted between October 2019 and July 2021. GPs in the intervention group followed an FCal-guided referral strategy and the control group adhered to Dutch GP guidelines, which do not recommend FCal testing. Eighty-four GP practices included 405 children aged 4–18 years with chronic abdominal pain and/or diarrhoea.

**Method:**

The primary outcome was specialist referral within 6 months after the baseline consultation. Incremental cost-effectiveness ratios (ICERs) were calculated from the societal perspective based on parental concern. Intention-to-treat (ITT) analyses and per-protocol (PP) analyses (strategy adherence in the intervention group and no FCal use in the control group) were conducted.

**Results:**

Adherence to the FCal strategy was 59%. Alarm symptoms for inflammatory bowel disease were more prevalent in the intervention group (26.6%) than the control group (8.9%). Referral rates were similar in the ITT analysis (22.8% versus 21.9%; adjusted odds ratio (AOR) = 0.94; 95% confidence interval [CI] = 0.57 to 1.54) and there were fewer referrals for the intervention group in the PP analysis (5.8% versus 20.3%; AOR = 0.21; 95% CI = 0.09 to 0.50). The intervention was not cost effective in the ITT analysis (ICER = €1534; 95% CI = -€9019 to €9579), but was cost effective in the PP analysis (ICER = -€344; 95% CI = -€4609 to €3096).

**Conclusion:**

The results do not justify recommending FCal testing in children in primary care without proper implementation. Additional research should focus on improving adherence to the FCal strategy.

## How this fits in

In children with chronic abdominal symptoms, GPs want to limit testing and referring of those with functional gastrointestinal disorders, but do not want to delay referral for those with organic disorders. Measuring faecal calprotectin (FCal) can add value to alarm symptoms and safely excludes inflammatory bowel disease (sensitivity ≥0.99). An FCal-guided referral strategy for children with chronic abdominal symptoms in primary care did not reduce referral rates (adjusted odds ratio = 0.94; 95% confidence interval = 0.57 to 1.54) and was not cost effective for reducing parental concern. Unfortunately, GPs only adhered to the strategy in 59% of children; however, when they did, referral rates were reduced and the strategy appeared to be cost effective.

## Introduction

Chronic abdominal symptoms are common in childhood and can have a major impact on both the child’s wellbeing and the wider healthcare system.^1^ In the Netherlands, children with these complaints will first consult a GP and ~90% will be diagnosed with a functional gastrointestinal disorder (FGID).^2–4^ However, it can be challenging to differentiate common FGID from comparatively rare, but severe, organic disorders, such as inflammatory bowel disease (IBD) or coeliac disease. Failure to recognise organic disorders may lead to treatment delay and complications,^5–9^ but the unnecessary referral of children with FGID to specialist care can leads to medicalisation,^10^ and incurs healthcare costs.^11^ Among children with chronic abdominal symptoms, 13-20% are referred to specialist care.^2,4,12^ This rate could be reduced given the low likelihood of severe organic disease (~1%)^13,14^ and the recommendation of the Dutch Society of GPs to treat children in general practice.^15^

When assessing children with abdominal pain, current Dutch GP guidelines recommend testing tissue transglutaminase immunogolobulin A (IgA) and total serum IgA for suspected coeliac disease, and testing haemoglobin, leukocytes, and the erythrocyte sedimentation rate (ESR) for suspected IBD.^15^ However, the tests for IBD have low or unknown sensitivity in primary care,^16–18^ and blood withdrawal is invasive and could be traumatic.^19^

Faecal calprotectin (FCal), a non-invasive marker of intestinal inflammation, might be useful for excluding IBD safely and reducing referrals for FGID. Studies have shown that FCal adds value to alarm symptoms in primary care settings, resulting in a sensitivity of ≥0.99 for levels of 50µg/g in children with chronic abdominal symptoms and alarm symptoms.^12,16,20,21^ Testing children without alarm symptoms increases the number of false positives to 13%^12^ and could lead to unnecessary referral to specialist care. The potential of FCal testing to increase referrals, coupled with its greater expense compared with currently recommended blood tests,^22^ necessitates an evaluation of the patient outcomes and costs associated with using FCal to inform referral decisions.

In this trial, the researchers assessed the (cost) effectiveness of an FCal-guided referral strategy in children with chronic abdominal symptoms compared with usual care (that is, adhering to Dutch GP guidelines, which do not recommend FCal testing).

## Method

### Design and setting

A pragmatic randomised controlled trial (RCT) was conducted in Dutch primary care; the process is outlined in Figure 1. Dutch GPs serve as the primary care provider for children and gatekeep referrals to specialist paediatric care. Cluster randomisation was chosen at the GP practice level to prevent contamination. Dutch GP practices were randomised by an investigator, who was not involved in the trial, using a computer-generated list with varying block sizes at a 1:1 ratio. GPs, children, and parents were not blinded to the intervention, but the research team was blinded during the statistical analyses.

**Figure 1.**
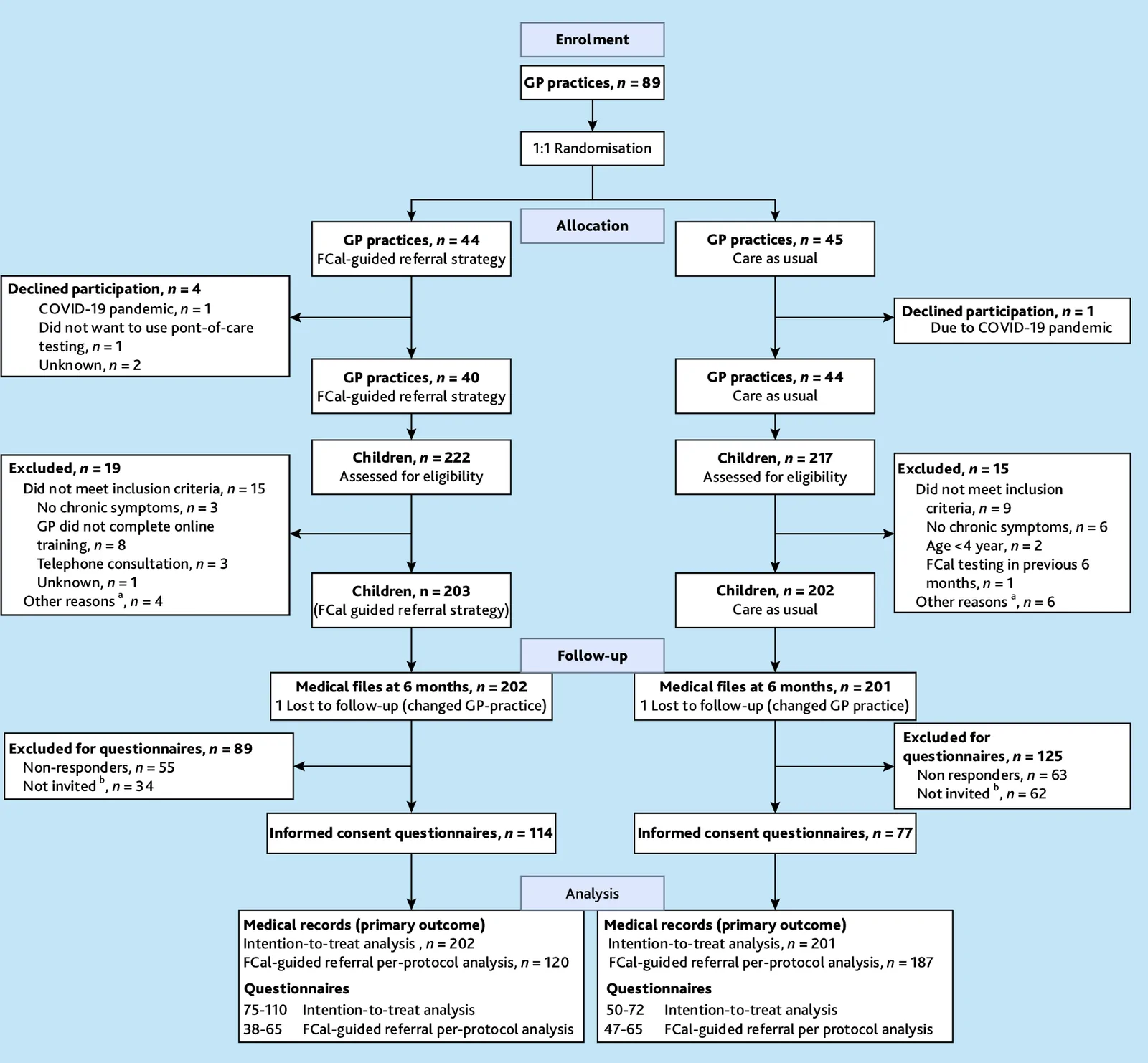
CONSORT flow diagram of the study process. ^a^Consultation preceding trial commencement (control, n=4; intervention, n=1), familiy problems (n=1 both arms), intervention group in another practice (control, n=1), no consent for sharing medical record (intervention, n=2). ^b^>3 months delay (control, n=57; intervention, n=26), language barrier (control, n=3; intervention, n=1), family problems (intervention, n=2), unknown (control, n=2; intervention, n=5).

A detailed description of the method has been published elsewhere^23^ and the statistical analysis plan, including a health economic analysis plan, is provided in Supplementary Information S1. The extended Consolidated Standards of Reporting Trials (CONSORT) statement for cluster RCTs^24^ and Consolidated Health Economic Evaluation Reporting Standards (CHEERS) 2022^25^ were followed.

All GP practices signed an agreement consenting to the collection of data from patient medical records. The Medical Research Ethics Committee (MREC) of University Medical Center Groningen waived the requirement to obtain written informed consent from participants for collecting data from patient medical records for the primary outcome, according to Dutch law (Medical Treatment Contracts Act). After obtaining informed consent from parents and/or children, questionnaires were used to collect information on secondary outcomes and costs; therefore, healthcare-use outcomes were assessed based on information from medical records in all included children, whereas questionnaire outcomes and costs were assessed using a subsample of consenting children.

### Participants

Children aged 4–18 years were eligible for inclusion if they had presented to a GP with:

- chronic abdominal pain (recurrent character for ≥2 months, or two or more episodes in the previous 2 months); and/or
- diarrhoea (soft-to-watery stool for ≥2 weeks, or two or more episodes in the previous 2 months).

Exclusion criteria were:

- an FCal test result in the preceding 6 months;
- referral to a paediatrician or endoscopy unit for gastrointestinal symptoms in the preceding 6 months; or
- history of chronic organic gastrointestinal disease.

Children were recruited during consultations and by email or telephone up to 3 months after a consultation. Each GP practice could include children over a 1-year period starting between 7 October 2019 and 2 December 2020; all data were collected on 12 January 2022.

### Interventions

GPs in the intervention group completed online training about the differential diagnosis of children with chronic abdominal symptoms, the alarm symptoms for IBD, the FCal strategy, and how to communicate a diagnosis of FGID. The FCal strategy entailed testing only those children with alarm symptoms and assessing the subsequent FCal result as follows:

- <50µg/g — consider IBD ruled out;
- 50–250µg/g — monitor the child; and
- >250µg/g — refer the child.

Due to the pragmatic nature of this trial, FCal use and referral decisions were left to the GPs’ discretion. FCal point-of-care-testing (POCT) devices were made available to GPs in the intervention group for use in their practices. The POCT has characteristics that are comparable to the standard laboratory test.^26–28^ In May 2020, the MREC approved a protocol amendment to change from POCT to standard laboratory FCal testing due to the possible risk of COVID-19 infection via stool samples^29,30^ and the shortage of personal protective equipment in Dutch GP practices. GPs in the control group provided care as usual, according to the guidelines of the Dutch Society of GPs, which recommend not testing FCal in children.^15,31–33^

### Outcomes

#### Effectiveness

The primary outcome was the proportion of children referred to specialist (secondary or tertiary) care within 6 months after their baseline consultation, as per the data extracted from patient medical records. The secondary outcomes — relating to healthcare use — were assessed 6 months after the baseline consultation and defined as:

- the proportion of children referred to specialist care with FGID;
- the proportion of children with at least one diagnostic investigation; and
- the proportion of children with at least one diagnostic investigation, other than FCal (that is ESR, leukocytes, haemoglobin, and C-reactive protein), for IBD.

Parental satisfaction with the baseline consultation was measured shortly thereafter (within 2 weeks), with the Parental Medical Interview Scale (P-MISS).^34^ The extent of parental concern and the child’s perceived health (based on the EQ-5DY)^35^ — both scored on a visual analogue scale (VAS) — as well as the impact of symptoms on the child’s daily function, based on the Functional Disability Inventory,^36^ were assessed via questionnaires completed shortly after the baseline consultation, and at 3 months and 6 months thereafter. The presence of chronic abdominal pain and/or diarrhoea was added as an outcome after the trial registration, as described in the protocol.^23^

#### Evaluation of costs

Costs were classified as follows:

- healthcare;
- patient and family; and
- other sectors.

Prices were based on the cost manual of the National Health Care Institute of the Netherlands^37^ and the standard prices of medications,^38^ indexed to 2022 and reported in euros^39^ (Supplementary Table 1). Healthcare costs were assessed for all children using medical records; patient and family costs, as well as other sector costs, were assessed only in consenting children based on adapted versions of the Institute of Medical Technology Assessment (iMTA) Medical Consumption Questionnaire^40^ and the iMTA Productivity Costs Questionnaire.^41^

### Statistical analyses

#### Effectiveness

##### Sample size

To detect a 10% ^2,12^ (from 17% to 7%) reduction in referrals, with a two-sided α of 5%, a power of 80%, an expected mean cluster size of three, an intraclass correlation coefficient of 0.06,^42,43^ and a loss to follow-up of 10%, the researchers calculated that 203 children were needed per study arm.

##### Missing data

For the primary and secondary outcomes that were assessed via medical records, missing data accounted for <5%, so imputation was not deemed necessary, as outlined by Heymans and Twisk.^44^ For the longitudinal data, missing data were not imputed because this is considered redundant.^45^

##### Main analyses

The primary outcome was analysed on an intention-to-treat (ITT) basis using multilevel multivariable regression analysis due to the hierarchal structure of the data. The GP practice was added as a random intercept, and both the intervention and the presence of alarm symptoms during the baseline consultation were added as covariates. The same random and fixed effects were used for the secondary outcomes. For longitudinal data, the child was added as a random effect, and both time and the interaction between time and the intervention were added as covariates. Parental concern before the baseline consultation was added as a covariate for the parental concern outcome.

All statistical tests were two-sided using a significance level of 0.05. Adjusted odds ratios (AORs) and adjusted β-coefficients with 95% confidence intervals (CIs) were reported. Analyses were carried out using SPSS Statistics (version 28).

##### Sensitivity analyses

Two per-protocol (PP) analyses were performed. In a prespecified PP analysis for FCal-guided referral, children from the intervention group were only included if the GP adhered to the indications for FCal testing (that is, to test only if alarm symptoms were present) and referral (no referral if the FCal was ≤50µg/g; referral if the FCal was >250µg/g). In a post-hoc PP analysis for FCal testing, all children with appropriate FCal testing were included (regardless of the GP’s referral behaviour), because the primary outcome (referral) was incorporated into the selection of children in the first PP analysis.

In both PP analyses, children in the control group were excluded if they received FCal testing. A post-hoc analysis was performed in which alarm symptoms were not adjusted for because a baseline difference between the study arms was observed.

### Evaluation of costs

#### Missing data

Comparing the characteristics and outcomes of children with, and without, complete cost–effect pairs suggested data were missing at random; therefore, multiple imputation on the level of aggregated costs per child (five times) via predictive mean matching was performed. The dataset following multiple imputation was used for the main analysis.

#### Main analysis

An incremental cost-effectiveness ratio (ICER) was calculated using mean parental concern as the effect parameter and measuring mean costs from a societal perspective (all costs) over a 6-month time horizon. As it was not normally distributed, bootstrapping (5000 resamples) was performed on the costs and the cost–effect pairs to produce CIs. Results of the bootstraps were used to generate cost-effectiveness planes, and cost-effectiveness acceptability curves (CEACs) were plotted.

#### Sensitivity analyses

In addition to the two PP analyses, two post-hoc sensitivity analyses were conducted: the first excluded children with IBD and the second used only complete cases.

### Patient and public involvement

The researchers collaborated with the Child and Hospital Foundation (*Stichting Kind en Ziekenhuis*), and incorporated their opinions and expertise in the grant proposal, patient information letters, recruitment strategies, and interpretation and dissemination of the study results. In addition, the study results were distributed to participating children and/or parents via a short e-mail newsletter.

## Results

### Participants

In total, 89 GP practices were recruited, primarily from the north east of the Netherlands. Of these, five declined to participate after randomisation; the remaining 84 were assigned to the intervention group (*n* = 40) or the control group (*n* = 44) (Figure 1). GPs enrolled 405 children between October 2019 and July 2021 (Figure 1). GP practices in the intervention group were slightly larger and more often located in urban areas than those in the control group (Table 1). More children in the intervention group than in the control group had IBD (*n* = 4 versus *n* = 0) and alarm symptoms (26.6% versus 8.9%) (Table 1). All children with IBD were referred during their first consultation. Overall, informed consent was provided for 114 (56.2%) and 77 (38.1%) children in the intervention and control groups respectively (Figure 1). Of these, 63 in the intervention group and 42 in the control group had complete data for cost–effect pairs.

**Table 1.** Baseline characteristics

	Intervention	Control	Intervention	Control
**GP practices**	***n* = 40**	***n* = 44**		
Practice type				
Solo, *n* (%)	13 (32.5)	18 (40.9)		
Duo, *n* (%)	16 (40.0)	14 (31.8)		
Group, *n* (%)	11 (27.5)	12 (27.3)		
Rural area^a^	17 (42.5)	27 (61.4)		
Distance to nearest hospital in km, median (IQR)	8.0 (3.8–16.2)	8.1 (3.8–16.5)		
Number of patients per practice, median (IQR)	3658 (2763–6458)	3152 (2418–4100)		
Number of children per practice, median % (IQR)	21.3 (20.0–24.6)	21.9 (20.0–23.7)		
Number of included children per practice, median (IQR)	4.0 (2.0–7.0)	3.0 (1.0–6.8)		
**GPs**	***n* = 127^b^**	***n* = 122^b^**		
Male sex, *n* (%)	55 (43.3)	54 (44.3)		
Experience in years, median (IQR)^c^	10.0 (3.0–19.0)	12.0 (3.0–21.0)		
Working days per week, median (IQR)	4.0 (3.0–4.5)	4.0 (3.0–4.0)		
Previous education on FCal in children, *n* (%)	7 (5.5)	10 (8.2)		
FCal use in children in the previous year, *n* (%)	33 (26.0)	33 (27.0)		
GP type, *n* (%)				
GP	110 (86.6)	100 (82.0)		
GP registrar	15 (11.8)	19 (15.6)		
Physician assistant	2 (1.6)	3 (2.5)		
**Children**	***n* = 203**	***n* = 202**	**Informed consent, *n* = 114**	**Informed consent, *n* = 77**
Male sex, *n* (%)	75 (36.9)	85 (42.1)	45 (39.5)	26 (33.8)
Age in years, median (IQR)	10.5 (7.5–14.0)	9.5 (7.2–14.0)	9.7 (7.0–13.5)	9.0 (7.0–12.9)
Symptoms, *n* (%)				
Chronic abdominal pain	161 (79.3)	157 (77.7)	92 (80.7)	59 (76.6)
Chronic diarrhoea	11 (5.4)	10 (5.0)	6 (5.3)	4 (5.2)
Chronic abdominal pain and diarrhoea	31 (15.3)	35 (17.3)	16 (14.0)	14 (18.2)
Alarm symptoms at baseline consultation	54 (26.6)	18 (8.9)	38 (33.3)	8 (10.4)
Positive family history for IBD	24 (11.8)	4 (2.0)	20 (17.5)	2 (2.6)
Rectal bleeding	14 (6.8)	6 (3.0)	8 (7.0)	3 (3.9)
Involuntary weight loss	13 (6.4)	4 (2.0)	7 (6.1)	2 (2.6)
Aphthous stomatitis	8 (3.9)	2 (1.0)	8 (7.0)	1 (1.3)
Decreased growth velocity	5 (2.5)	3 (1.5)	3 (2.6)	2 (2.6)
Perianal abnormalities	5 (2.5)	1 (0.5)	4 (3.5)	0 (0.0)
Skin abnormalities	3 (1.5)	1 (0.5)	1 (0.9)	0 (0.0)
Arthritis	0	0	0	0
Eye inflammation	0	1	0	0
>1 alarm symptom	14 (6.9)	4 (2.0)	10 (8.8)	2 (2.6)
Recruited by GP during consultation, *n* (%)	100 (49.3)	69 (34.2)	69 (60.5)	35 (45.5)
FCal use baseline consultation, *n* (%) Point-of-care testing, *n* (%)	63 (31.0) 29 (14.3)	10 (5.0) 0 (0.0)	39 (34.2) 19 (16.7)	6 (7.8) 0 (0.0)
FCal result, *n* (%)				
<50µg/g	48 (23.6)	10 (5.0)	32 (28.1)	6 (7.8)
50–250μg/g	10 (4.9)	0	3 (2.6)	0
>250µg/g	5 (2.5)	0	4 (3.5)	0
Diagnosis – 6-month follow-up, *n* (%)				
FGID^d^	176 (86.7)	186 (92.1)	100 (87.7)	71 (92.2)
Gastroenteritis	8 (3.9)	7 (3.5)	3 (2.6)	4 (5.2)
Gastroesophageal reflux	4 (2.0)	0	1 (0.9)	0
Food/lactose intolerance^e^	5 (2.5)	4 (2.0)	3 (2.6)	1 (1.3)
IBD	4 (2.0)	0	3 (2.6)	0
Coeliac disease	0	0	0	0
Other^f^	5 (2.5)	3 (1.5)	4 (3.5)	1 (1.3)
Lost-to-follow-up	1 (0.5)	2 (1.0)	0	1 (1.3)

a Rural area is defined as <500 addresses/km^2^.^64,65^

b Number of children included in analysis ranged from 121 to 127 for the intervention group and from 109 to 122 for the control group.

c All GP trainees are considered to have 0 years’ experience.

d Diagnosis at 6-month follow-up was based on GP’s or medical specialist’s documentation about the diagnosis (text or International Classification of Primary Care codes).

e Comprises functional abdominal pain, functional constipation, irritable bowel syndrome, a combination of the three, abdominal pain with unknown reason, and diarrhoea with unknown reason.

f Independent of whether appropriate testing (for example, a hydrogen breath test) was performed.

g Intervention group: systemic mastocytosis (*n *= 1), pin worms (*n *= 1), mesenteric lymphadenitis (*n *=1), Helicobacter pylori gastritis (*n *= 1), and dysmenorrhoea (*n *= 1). Control group: familial Mediterranean fever (*n *= 1), recurring urinary tract infections (*n *= 1), and dysmenorrhoea (*n *= 1).

FCal = faecal calprotectin; FGID = functional gastrointestinal disorder; IBD = inflammatory bowel disease; IQR = interquartile range;

### ITT analyses

The crude referral rate was comparable in both study groups: 22.8% and 21.9% in the intervention and control groups respectively (Table 2). Multilevel analysis with adjustment for alarm symptoms and GP practice showed no difference in referral rates (AOR = 0.94; 95% CI = 0.57 to 1.54) (Table 2). No clinically significant differences were found between the study groups for secondary outcomes (Table 2). The sensitivity analysis, without correcting for alarm symptoms, also showed no effect on the referral rate (AOR = 1.05; 95% CI = 0.66 to 1.68) or the secondary outcomes (Table 2).

**Table 2.** Outcomes in intention-to-treat population

		Intervention, *n* = **203**	Control, *n* = **202**		AOR/adjusted β coefficient (95% CI)^a^	AOR/adjusted β coefficient (95% CI)^b^
	***n***	**Median (IQR) or *n* (%)**	***n***	**Median (IQR) or *n* (%)**		
Referral to specialist care	202	46 (22.8)	201	44 (21.9)	0.94 (0.57 to 1.54)	1.05 (0.66 to 1.68)
Referral to specialist care and FGID diagnosis	202	36 (17.7)	201	38 (18.8)	0.89 (0.53 to 1.50)	0.93 (0.56 to 1.54)
Diagnostic investigation for IBD **^c^**	202	61 (30.2)	201	62 (30.8)	0.82 (0.49 to 1.39)	0.98 (0.61 to 1.59)
Diagnostic investigation	202	140 (69.3)	201	110 (54.7)	1.59 (0.92 to 2.76)	1.93 (1.15 to 3.24)
Parental satisfaction about baseline consultation **^d^**	106	4.10 (3.68–4.50)	71	4.19 (3.81–4.50)	-0.08 (-0.27 to 0.12)	-0.05 (-0.23 to 0.15)
**Degree of parental concern (VAS)** ^e^						
Before baseline consultation	100	4.45 (2.50–6.00)	68	3.85 (2.50–6.00)	-	-
Shortly after baseline consultation	97	3.70 (2.05–5.00)	68	3.10 (1.93–5.00)	0.35 (-0.34 to 1.04)	0.30 (-0.38 to 0.98)
At 3 months	93	3.50 (1.90–6.10)	63	3.60 (1.70–6.00)	-0.08 (-0.82 to 0.66)	-0.14 (-0.87 to 0.59)
At 6 months	85	3.00 (1.30–5.70)	58	2.85 (1.50–5.28)	-0.10 (-0.87 to 0.67)	-0.15 (-0.91 to 0.61)
**Presence of chronic diarrhoea** ^f^						
Shortly after baseline consultation	110	62 (56.4)	71	45 (63.4)	0.62 (0.26 to 1.49)	0.70 (0.30 to 1.61)
At 3 months	103	40 (38.8)	68	30 (44.1)	0.66 (0.27 to 1.61)	0.74 (0.32 to 1.74)
At 6 months	91	38 (41.8)	64	24 (37.5)	1.05 (0.42 to 2.65)	2.10 (1.10 to 4.01)
**Presence of chronic abdominal pain** ^g^						
Shortly after baseline consultation,	93	68 (73.1)	66	47 (71.2)	1.08 (0.46 to 2.54)	1.08 (0.47 to 2.48)
At 3 months	86	46 (53.5)	54	18 (33.3)	2.59 (1.08 to 6.24)	2.57 (1.09 to 6.04)
At 6 months	75	24 (32.0)	50	18 (36.0)	0.80 (0.32 to 2.03)	0.80 (0.32 to 1.98)
**Health (VAS)**						
Shortly after baseline consultation	103	8.00 (6.50–9.00)	67	8.00 (6.50–8.50)	0.05 (-0.49 to 0.58)	0.03 (-0.50 to 0.55)
At 3 months	97	8.00 (7.30–8.80)	64	8.05 (7.03–8.85)	0.04 (-0.50 to 0.58)	0.03 (-0.50 to 0.55)
At 6 months	86	8.40 (7.30–9.23)	61	8.10 (7.35–9.00)	0.05 (-0.50 to 0.60)	0.04 (-0.50 to 0.58)
**Impact symptoms on child’s daily function** ^h^						
Shortly after baseline consultation	110	0.43 (0.13–0.88)	72	0.37 (0.13–0.67)	0.17 (-0.01 to 0.34)	0.18 (0.02 to 0.35)
At 3 months	103	0.27 (0.00–0.73)	70	0.27 (0.05–0.62)	0.06 (-0.12 to 0.23)	0.07 (-0.10 to 0.24)
At 6 months	86	0.20 (0.00–0.48)	65	0.20 (0.00–0.47)	0.01 (-0.17 to 0.19)	0.03 (-0.15 to 0.20)

a Adjusted for GP practice and alarm symptoms.

b Adjusted for GP practice.

c Erythrocyte sedimentation rate, haemoglobin, leukocytes, and C-reactive protein.

d Based on the Parental Medical Interview Scale, 26 items on a Likert scale from ‘very unsatisfied’^1^ to ‘very satisfied’.^5^

e Both βs are adjusted for parental concern before baseline consultation.

f Soft-to-watery stool for ≥2 weeks or two or more episodes in the previous 3 months.

g Abdominal pain or discomfort with a recurrent character for ≥2 months or two or more episodes in the previous 3 months. Due to a technical fault in the online questionnaire, data were missing completely at random for some participants.

h Functional Disability Inventory, 15 daily activities scored on a five-point scale from ‘no trouble’ (0) to ‘impossible’.^4^

AOR = adjusted odds ratio; CI = confidence interval; FGID = functional gastrointestinal disorder; IBD = inflammatory bowel disease; IQR = interquartile range; OR = odds ratio; VAS = visual analogue scale;

### PP analyses

GPs did not adhere to the FCal strategy for 82 out of 202 children (41%) (Figure 2); reasons were FCal testing in children without alarm symptoms (*n* = 31), no FCal testing in children with alarm symptoms (*n* = 28), and referral with a negative or absent FCal value (*n* = 23) (Figure 2).

**Figure 2.**
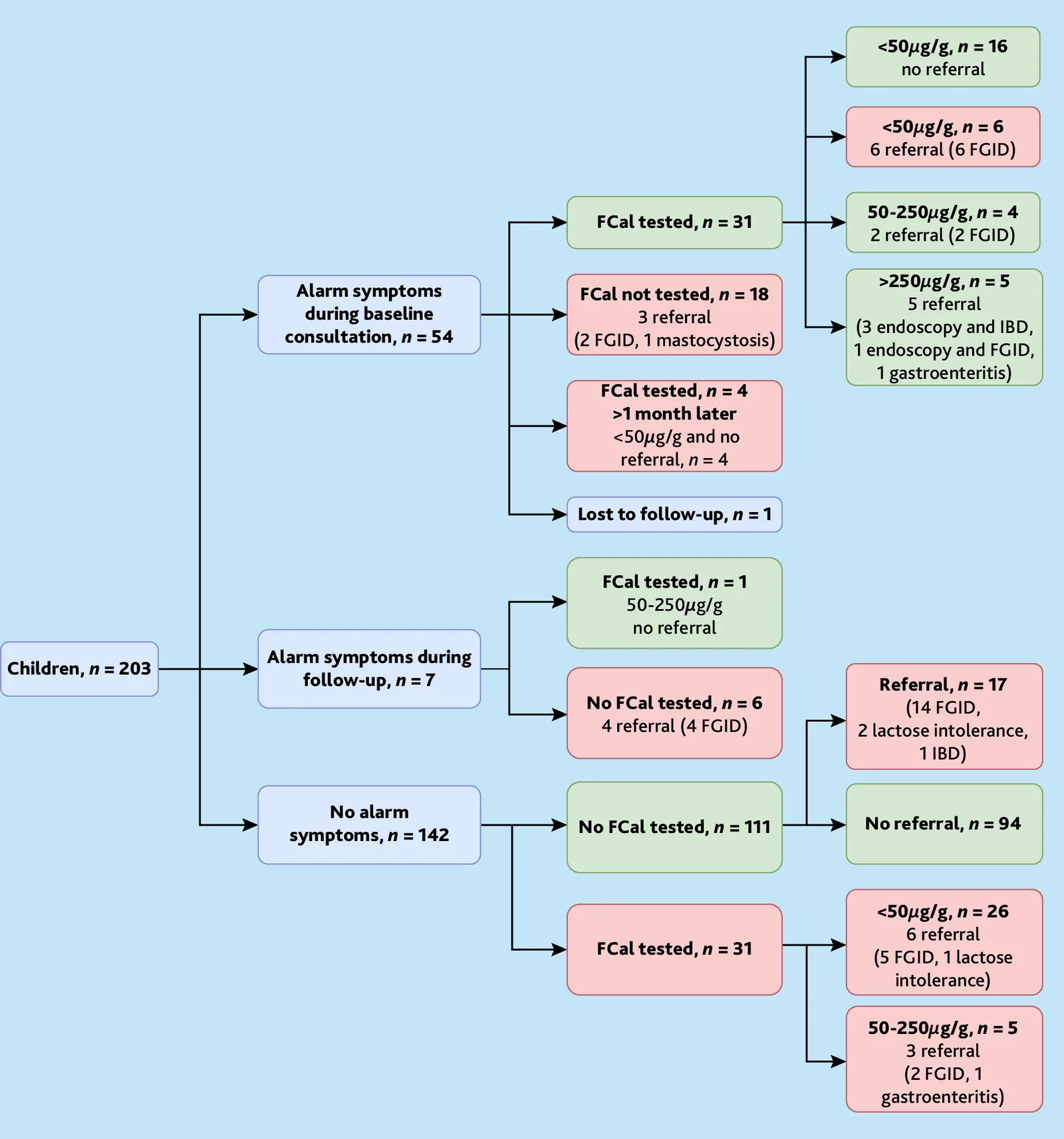
GPs’ adherence to the FCal-guided referral strategy in the intervention group. ^a^Green boxes reflect children treated in line with the FCal guided referral strategy; red boxes reflect children not treated in line with the FCal guided referral strategy. FCal = faecal calprotectin; FGID = functional gastrointestinal disease; IBD = inflammatory bowel disease.

The PP analysis for FCal-guided referral included 120 (59%) children from the intervention group and 187 (93%) children from the control group (Figure 1, Supplementary Tables 2 and 3). This revealed a persistent imbalance in reported alarm symptoms, with referral rates of 5.8% and 20.3% for the intervention and control groups respectively (AOR = 0.21; 95% CI = 0.09 to 0.50) (Supplementary Tables 1 and 2). Differences remained when not correcting for alarm symptoms (AOR = 0.24; 95% CI = 0.10 to 0.57) (Supplementary Table 3). The proportion of children referred to specialist care with FGID was lower in the intervention group (2.5%) than in the control group (18.2%) (AOR = 0.17; 95% CI = 0.06 to 0.46) (Supplementary Table 3), which remained when not correcting for alarm symptoms (AOR = 0.20; 95% CI = 0.08 to 0.53). Other secondary outcomes were similar between study groups (Supplementary Table 3).

In the PP analysis for FCal testing, no clinically significant differences existed between the two study groups (AOR for primary outcome = 0.86, 95% CI = 0.49 to 1.51) (Supplementary Tables 4 and 5).

### Evaluation of costs

Total mean costs per child in the intervention group (€425) were higher than in the control group (€323), which was mainly driven by differences in the costs of parents’ work absenteeism (Table 3). To achieve a unit improvement on the parental concern VAS after 6 months, the additional costs of the FCal strategy were €1534 per child (95% CI = -€9019 to €9579) (Supplementary Table 6). The cost-effectiveness plane revealed that most bootstrap replicates surrounded the centre of the plane, indicating uncertainty as to whether the effects and costs favoured the intervention or control (Figure 3). The CEAC showed a relatively low maximum probability of cost effectiveness (0.64), which was reached at a cost-effectiveness threshold of approximately €5000 per unit improvement (Supplementary Figure 1).

**Figure 3.**
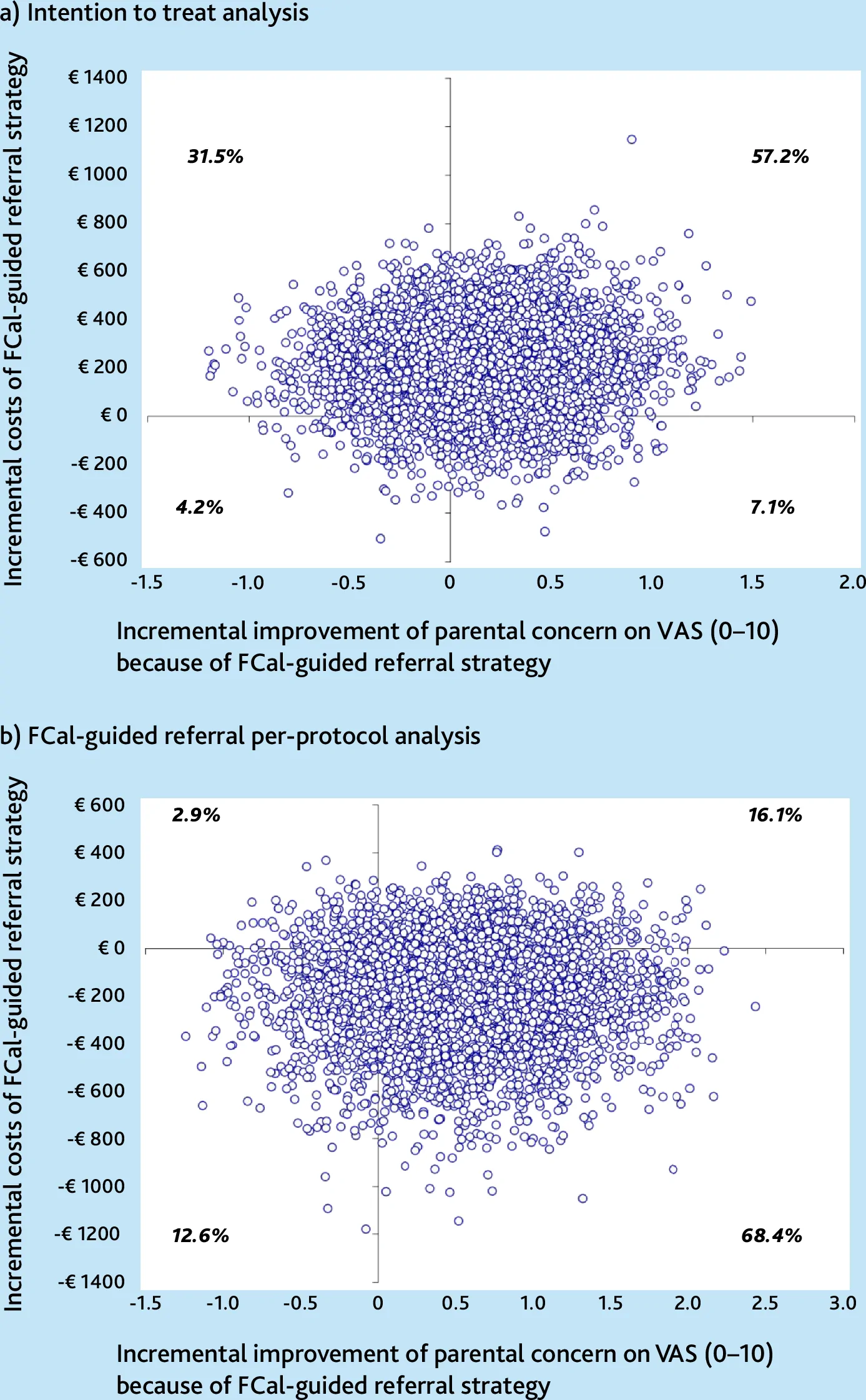
**a)** Cost-effectiveness planes of the intention-to-treat analysis. **b)** Cost-effectiveness planes of the FCal-guided referral per-protocol analysis. FCal = faecal calprotectin; VAS = visual analogue scale.

**Table 3.** Mean costs for the intervention and control groups

	Intervention, € (95% CI)	Control, € (95% CI)
**Non-healthcare costs**	***n* = 78^a^**	***n* = 52^a^**
**Patients and family**		
Over-the-counter medication	3 (1 to 7)	1 (0 to 2)
Diet^b^	23 (10 to 40)	19 (9 to 29)
Alternative treatments^c^	11 (3 to 22)	11 (0 to 25)
Incontinence costs^d^	3 (0 to 8)	4 (1 to 8)
Day care	0	0
*Subtotal*	*38 (22 to 58)*	*32 (16 to 53)*
**Other sectors**		
Work absenteeism of parents	184 (53 to 368)	85 (6 to 217)
Informal care by family and friends	202 (57 to 391)	230 (41 to 597)
School absence child, days	–	–
*Subtotal*	*340 (111 to 664)*	*300 (82 to 609)*
**Healthcare costs**	***n* = 202**	***n* = 201**
Primary care		
GP consultation	58 (55 to 62)	50 (48 to 52)
Other primary healthcare professionals^e^	11 (5 to 17)	9 (4 to 15)
Diagnostic investigations	84 (67 to 102)	81 (63 to 100)
Blood tests	18 (14 to 22)	15 (11 to 20)
FCal POCT	6 (4 to 8)	–
FCal laboratory tests	11 (8 to 14)	4 (2 to 6)
Faeces other tests	41 (26 to 57)	56 (39 to 73)
Urine tests	1.1 (0 to 2)	1.3 (1 to 2)
Other^f^	6 (3 to 10)	5 (2 to 7)
Prescribed medication	15 (11 to 19)	15 (11 to 21)
Specialist care		
Consultation with medical specialist	63 (45 to 82)	46 (33 to 60)
FCal laboratory	4 (2 to 6)	4 (2 to 6)
Endoscopy	9 (2 to 21)	3 (0 to 9)
Other hospital costs^g^	15 (4 to 31)	11 (0 to 26)
*Subtotal*	*259 (221 to 302)*	*220 (189 to 253)*
**Total**	**425 (305 to 583)**	**323 (242 to 423)**

a Complete cost–effect pairs: intervention, *n *= 63/78; control, *n *= 42/52.

b Adjusted diet and diet supplements.

c Alternative medication and treatments.

d Nappies, mattresses, laundry.

e Physical therapy, psychotherapy, and dietician within the primary care system.

f Emergency room visits and hospital admissions.

g Abdominal ultrasound, abdominal X-ray, and tests for sexually transmitted diseases.

FCal = faecal calprotectin; POCT = point-of-care testing;

In the PP analysis for FCal-guided referral, the ICER favoured the intervention (ICER = -€344 [95% CI = -€4609 to €3096]), indicating that €344 was saved per child for every unit improvement on the parental concern VAS after 6 months. This was driven by lower costs in the intervention group. The cost-effectiveness plane revealed 68.4% of the bootstrap replicates in the bottom-right quadrant, indicating lower costs and better effects with FCal-guided referral (Figure 3). The CEAC indicated there was a maximum probability of 0.81 that the intervention was cost effective without investing additional money; however, at an investment of approximately €1000 per unit improvement, the probability of the intervention being cost effective increased to 0.86 (Supplementary Figure 1). Other sensitivity analyses revealed results that were comparable to those of the main analysis (Supplementary Figure 2).

## Discussion

### Summary

This cluster RCT in primary care showed that an FCal-guided referral strategy for children with chronic abdominal symptoms did not reduce referrals to specialist care and was not cost effective. When assessed using the 59% of cases in which GPs adhered to all steps in the strategy, referrals were reduced and the strategy appeared to be cost effective; however, no intervention effect was observed in cases with appropriate FCal testing, regardless of the subsequent referral management. The results were similar regardless of adjustment for alarm symptoms.

### Strengths and limitations

The trial presented here evaluated the impact of FCal testing on management decisions and patient outcomes in routine daily practice. Such a diagnostic impact study contributes to providing high-quality evidence about diagnostic tests.^46,47^ Although pragmatic trials have the potential to mask any real effect due to variation, the outcomes are both relevant to decision makers and generalisable to real-world-settings,^48,49^ especially in those where GPs or primary care paediatricians function as a gatekeeper to specialist care.^48,49^

The slight imbalance in GP practice size and location between the two groups is a common observation in clustered RCTs,^50^ but is not expected to cause significant confounding because differences between GP practices were accounted for by estimating a random intercept. Another limitation of cluster RCTs is post-randomisation differential recruitment that causes selection bias;^51^ indeed, more alarm symptoms were observed among children in the intervention group compared with the control group, which is probably a result of greater awareness of alarm symptoms in the intervention group, given that other child characteristics were comparable. The higher occurence of alarm symptoms in the intervention group may have underestimated the effect of FCal testing as GPs were more often alerted, and may have tested and referred more children as a result. Finally, the statistical power for the patient-reported outcomes was low due to the informed consent requirement.

Referral was not chosen as the effect parameter for the ICERs, as referral rates would have been reflected in both the costs and the effects of the ICERs. The subjective measure of parental concern was chosen for the cost-effectiveness analysis because the researchers thought that would drive a GP’s further management through, for example, diagnostic testing and referral. Quality-adjusted-life-years (QALYs) were not measured, due to the lack of available tariff for children at the moment of designing this study. In addition, although QALYs are formally used in the UK, in many other countries their use is not a formal procedure.

### Comparison with existing literature

This is, to the authors’ knowledge, the first RCT to evaluate the (cost) effectiveness of an FCal-guided referral strategy for children with chronic abdominal symptoms in primary care. The findings contradict the results of an observational study that revealed a 28% reduction in referrals to specialist care following the implementation of an FCal-based referral pathway in primary care.^21^ That earlier study relied on a comparison between actual referrals and whether GPs would have referred without knowing the FCal result^21^ — a distinction that may have led to an overestimation of the intervention effect. Nevertheless, in that earlier study, no difference was found in the time to diagnosis for IBD among children with FCal testing compared with direct referral to specialist care.^21^ The researchers of the study presented here did not evaluate the time to diagnose IBD due to its low prevalence in primary care,^13^ although the intervention is not expected to delay diagnosis because GPs were instructed about alarm symptoms and FCal sensitivity exceeds that of currently recommended blood tests.^12,16–18,20,21^ It is unlikely that cases of IBD were missed because most will typically be referred within 6 months of presentation.^52,53^

GPs’ adherence to the FCal strategy was poor. The finding that some GPs still referred children with a negative FCal result aligns with results from tertiary care showing that paediatric gastroenterologists still performed endoscopies in children with negative FCal.^54^ It could be difficult for GPs to use a probabilistic output in diagnostic testing given that their clinical decision making is usually more intuitive than analytical.^55^ GPs not adhering to the test and referral indications might have motivations other than excluding organic disease, such as parental reassurance, being seen to be doing something, or to seek specialist help for the management of FGID.^56^,^57–59^

Previous model-based studies showed that FCal testing could be cost effective in guiding treatment decisions for adults and children in primary care.^60–62^ The study presented here is, to the authors’ knowledge, the first to use real-world data to evaluate FCals cost effectiveness, and only showed cost effectiveness in the PP analysis.

Failure to improve parental concern to a considerable degree in the intervention group is consistent with previous literature that showed that diagnostic testing alone does not reassure patients^63–65^ and, often, has a minimal impact on patient-reported outcomes, such as quality of life.^66^

### Implications for research

Although FCal testing adds high diagnostic value to alarm symptoms, this did not translate to fewer referrals, lower reliance on other diagnostic tests, improved patient-reported outcomes, or lower costs. PP analyses showed that the correct use of the FCal-guided referral strategy could not only reduce referrals, but could do so in a cost-effective manner. However, low strategy adherence by GPs in daily practice would mean that, even if the test was incorporated into guidelines with clear instructions, improper use would likely prevent improved care. Future research should aim to disentangle the key factors that lead to diagnostic tests and referrals in children with chronic abdominal symptoms, and improved adherence to diagnostic strategies.

## Data

Meta data is publicly available: DOI: https://doi.org/10.34760/6748340658518
